# Perinatal Exposure to Environmentally Relevant Levels of Bisphenol A Decreases Fertility and Fecundity in CD-1 Mice

**DOI:** 10.1289/ehp.1002559

**Published:** 2010-12-02

**Authors:** Nicolas J. Cabaton, Perinaaz R. Wadia, Beverly S. Rubin, Daniel Zalko, Cheryl M. Schaeberle, Michael H. Askenase, Jennifer L. Gadbois, Andrew P. Tharp, Gregory S. Whitt, Carlos Sonnenschein, Ana M. Soto

**Affiliations:** 1 Department of Anatomy and Cellular Biology, Tufts University School of Medicine, Boston, Massachusetts, USA; 2 Institut National de la Recherche Agronomique, UMR1089 Xénobiotiques, Toulouse, France

**Keywords:** biphasic dose response, bisphenol A, endocrine disruptor, fecundity, fertility, fetal origins of adult disease, perinatal exposure

## Abstract

**Background:**

Perinatal exposure to low-doses of bisphenol A (BPA) results in alterations in the ovary, uterus, and mammary glands and in a sexually dimorphic region of the brain known to be important for estrous cyclicity.

**Objectives:**

We aimed to determine whether perinatal exposure to environmentally relevant doses of BPA alters reproductive capacity.

**Methods:**

Female CD-1 mice that were exposed to BPA at 0, 25 ng, 250 ng, or 25 μg/kg body weight (BW)/day or diethylstilbestrol (DES) at 10 ng/kg BW/day (positive control) from gestational day 8 through day 16 of lactation were continuously housed with proven breeder males for 32 weeks starting at 2 months of age. At each delivery, pups born to these mating pairs were removed. The cumulative number of pups, number of deliveries, and litter size were recorded. The purity of the BPA used in this and our previous studies was assessed using HPLC, mass spectrometry, and nuclear magnetic resonance.

**Results:**

The forced breeding experiment revealed a decrease in the cumulative number of pups, observed as a nonmonotonic dose–response effect, and a decline in fertility and fecundity over time in female mice exposed perinatally to BPA. The BPA was 97% pure, with no evidence of contamination by other phenolic compounds.

**Conclusions:**

Perinatal exposure to BPA leads to a dose-dependent decline in the reproductive capacity of female mice. The effects on the cumulative number of pups are comparable to those previously reported in mice developmentally exposed to DES, a compound well known to impair reproduction in women. This association suggests the possibility that early BPA exposure may also affect reproductive capacity in women.

Bisphenol A (BPA) has received heightened attention in the last decade because of its ubiquitous presence. BPA is used in the production of polycarbonate plastics and epoxy resins, including the resins used to coat the inside of food and beverage cans, and for dental composites and sealants (Olea et al. 1996; Vandenberg et al. 2007; Ye et al. 2009). A growing body of evidence supports the chronic nature of human BPA exposure. BPA was detected in the urine of > 92% of Americans tested, with higher levels present in children and adolescents relative to adults (Calafat et al. 2008). BPA has also been detected in maternal (Padmanabhan et al. 2008) and fetal plasma, placenta, and amniotic fluid (Ikezuki et al. 2002; Schönfelder et al. 2002) and in breast milk (Sun et al. 2004). The levels of urinary BPA in premature infants in neonatal intensive care units are approximately 10 times higher than those in the general population (Calafat et al. 2009).

BPA has been considered to be a weak estrogen because of its low potency compared with estradiol in assays involving nuclear receptors (Blair et al. 2001; Thomas and Dong 2006). However, low levels of BPA can act additively with other xenoestrogens as well as with natural estrogens (Silva et al. 2002; Soto et al. 1994, 1997; Tollefsen 2002). BPA can also act via membrane receptors to produce effects that are similar in potency to those of estradiol (Welshons et al. 2006; Wozniak et al. 2005). These findings stress the relevance of low-dose exposures, particularly during the perinatal period, when BPA may affect the development of estrogen target organs.

Developmental exposure to endocrine disruptors has been postulated to play a role in the increased incidence of malformations of the male genital tract and testicular and breast cancers observed in the European and U.S. populations during the last half century, a time when numerous hormonally active chemicals were introduced into the environment (Diamanti-Kandarakis et al. 2009; Jouannet et al. 2001; Skakkebaek 1998; Skakkebaek et al. 2001). Epidemiological studies of women born in Denmark have suggested that young cohorts had progressively lower total fertility rates that were compensated for by the increased use of assisted reproductive technologies (Jensen et al. 2008). In a laboratory setting, female mice exposed *in utero* to environmentally relevant doses of BPA have shown advanced puberty (Howdeshell et al. 1999), alterations in estrous cyclicity, and an increase in the percentage of ovarian antral follicles (Markey et al. 2003). Early BPA exposure resulted in increased proliferative activity of epithelial cells in the endometrial glands and increased expression of estrogen receptor-α (ERα) and progesterone receptor in the luminal epithelium of the endometrium and in the subepithelial stroma of exposed mice (Markey et al. 2005). Perinatal exposure of mice to BPA also decreased sex differences in the anteroventral periventricular nucleus of the hypothalamus (Rubin et al. 2006), a nucleus known to be important for the preovulatory luteinizing hormone (LH) surge. In addition, BPA exposure during gestation and lactation accelerated the onset of acyclicity in rats (Rubin et al. 2001). Taken together, these multiple effects of BPA exposure during early development might be expected to compromise overall reproductive success.

The above-mentioned effects observed after early exposure to BPA are reminiscent of those observed after fetal exposure to another estrogenic compound, diethylstilbestrol (DES), which was widely prescribed to prevent miscarriages in pregnant women (Newbold 2008). Fetal exposure to DES resulted in oviduct malformations in mice during adulthood (Newbold et al. 1983) and uterine fibroids in women later in life (Baird and Newbold 2005; McLachlan et al. 1980). Early exposure to DES also advanced the age of vaginal opening and the age at first estrous in mice (Honma et al. 2002). In addition, prenatal exposure to DES altered the reproductive capacity of female mice in a forced breeding protocol (McLachlan et al. 1982). This protocol revealed significant effects of DES, even at low doses, in stark contrast with the lack of effect of DES reported in studies that examined only the first pregnancy in DES-exposed females (Honma et al. 2002).

Based on the wide variety of effects on the reproductive system caused by fetal and/or neonatal exposure to BPA, and the reproductive effects caused by early exposure to DES, we hypothesized that perinatal exposure to BPA impairs the reproductive function of exposed females (Maffini et al. 2006). In the present study, we tested this hypothesis by assessing fertility and fecundity in BPA-exposed CD-1 mice over a period of 32 weeks in a forced breeding regimen.

## Materials and Methods

### Chemicals

Dimethyl sulfoxide (DMSO; CAS no. 67-68-5), DES (CAS no. 56-53-1), and BPA (4,4′-dihydroxydiphenyl dimethylmethane; CAS no. 80-05-7; product no. 23965-8, lot no. 03105ES; purity ≥ 99%) were purchased from Sigma Chemical Company (Saint Louis, MO, USA). We further analyzed the purity of the BPA stock by HPLC using a Spectra P1000 pump (Thermo Separation Products, Les Ulis, France) associated with a 250 mm × 4.6 mm (5 μm) Zorbax SB-C_18_ column protected by a Kromasil C_18_ guard precolumn (Agilent; Interchim, Montluçon, France). Mobile phases and analytical conditions were as previously described (Jaeg et al. 2004). The system was coupled to a diode area detector (UV6000LP Spectra System; wavelength range, 220–360 nm; Thermo Separation Products). Next, the mass spectrometric (MS) analysis of BPA was performed on a quadruple ion trap LCQ mass spectrometer (Thermo Electron, Les Ulis, France) equipped with an electrospray ionization (ESI) source. Finally, the nuclear magnetic resonance (NMR) spectrum of BPA was obtained at 300K on a Bruker Avance DRX-600 spectrometer (Bruker, Wissembourg, France) operating at 600.13 MHz and equipped with a 5-mm H, C, N inverse triple-resonance TXI cryoprobe attached to a cryoplatform (the preamplifier cooling unit). BPA (1 mg) was dissolved in 600 μL deuterated methanol (CD_3_OD), and one-dimensional spectrum was acquired using a standard pulse sequence for ^1^H NMR. Sixteen free induction decays were collected with a spectral width of 12 ppm into 64,000 data points.

### Animals

CD-1 female mice (12 weeks of age) and proven breeder male mice (all from Charles River Laboratories, Wilmington, MA, USA) were maintained in temperature- and light-controlled (14/10-hr light/dark cycle) conditions at the at Tufts University Human Nutrition and Research Center animal facility (approved by the Association for Assessment and Accreditation of Laboratory Animal Care International). All experimental procedures were approved by the Tufts University–New England Medical Center Institutional Animal Care and Use Committee. The animals were treated humanely and with regard for alleviation of suffering. Cages, water, and bedding all tested negligible for estrogenicity by the E-SCREEN assay (Soto et al. 1992). Water was supplied *ad libitum* from glass bottles. Food (Teklad 2018; Harlan, Indianapolis, IN, USA) was supplied *ad libitum*. The estrogenicity of food lots used was measured by the E-SCREEN assay and found to be negligible (< 20 pmol estrogen equivalents per gram of feed). The estrogenic activity of the feed was also tested independently by W.V. Welshons in his laboratory (Welshons et al. 1990).

Mice were allowed to acclimatize for 5 days before being paired to mate. The morning on which a vaginal plug was detected was considered gestational day (GD) 1. On the evening of GD8, dams were implanted subcutaneously with Alzet osmotic pumps (model 1004; Alza Corp., Palo Alto, CA, USA) following the manufacturer recommendations. Pumps were designed to deliver vehicle alone (50% DMSO in water), the positive control (DES), or one of three doses of BPA. These pumps continued to release at a constant rate (0.11 μL/hr) until day 16 of lactation. The actual delivered dose of BPA decreased as pregnancy progressed because the weight of the mother at GD6 was used to calculate BPA dose, and body weights (BW) increased from this point throughout pregnancy. BPA exposure groups were 0 (control), 25 ng, 250 ng, and 25 μg BPA/kg BW/day. DES was administered at 10 ng/kg BW/day, which was the lowest effective dose tested in a forced breeding regimen (McLachlan et al. 1982). The dams delivered naturally, and the F_1_ litters were culled to eight pups on the day after birth, keeping at least two males. Litters were weaned on postnatal day 21.

### Experimental design

At 8 weeks of age, one F_1_ female mouse from each treatment group was randomly chosen from each litter and individually housed with a nonexposed young male of proven fertility for a period of 32 weeks. A total of 18–21 dams in each group delivered litters (control, *n* = 21; BPA 25 ng/kg, *n* = 19; BPA 250 ng/kg, *n* = 18; BPA 25 μg/kg, *n* = 20; DES, *n* = 20). All the breeding cages were examined daily. On the day of each delivery, F_2_ pups were separated from the mother, and their number, sex, and weight were recorded. The pups were sacrificed by decapitation, and the F_1_ dam was immediately returned to the breeding cage. The cumulative number of live pups delivered, time to first litter, number of deliveries (fertility) per dam, number of neonates per litter (fecundity), and number of mice having litters were recorded.

### Statistics

We used SPSS statistical software (version 15.0; SPSS Inc., Chicago, IL, USA) for all statistical analyses. The Kolmogorov-Smirnov test was used to assess differences in the cumulative number of pups between treatment groups. Differences in the total number of pregnancies were assessed using analysis of variance (ANOVA) and Dunnett’s post hoc test. Fisher’s exact test was used to analyze differences in the proportions of female mice in each treatment group with four or more pregnancies. All these results are presented as mean ± SE or percentages. To study fecundity, scatter plots and trend lines of the number of pups versus delivery number were produced for each treatment group. The slope and correlation coefficient (*R*) of the trend lines were each compared across treatment groups using *t*-tests. For all statistical tests, results were considered significant at *p* < 0.05.

## Results

### Purity of BPA

**Figure 1:**
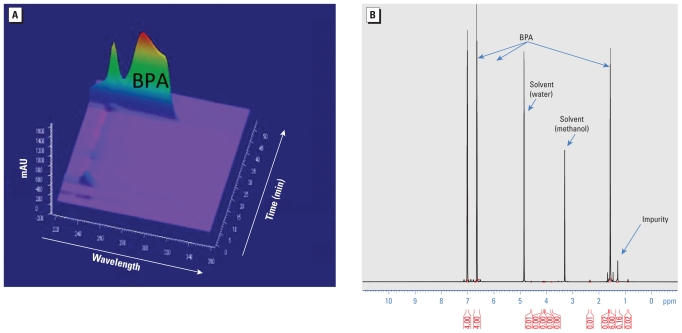
Diode area detector HPLC-UV analysis (*A*) and 600 MHz ^1^H NMR spectrum (*B*) of the BPA used in these experiments (Sigma Aldrich catalog no. 23965-8).

When analyzed by HPLC with ultraviolet (UV) detection, the retention time of our BPA stock (43 min) was identical to that expected for authentic BPA (Zalko et al. 2003). Based on UV absorbance at 269 nm, BPA purity was close to 100% (Figure 1A). We detected minor impurities at lower wavelengths that eluted from the column with retention times of 10 min (major peak) and 22–23 min (minor peak), respectively. ESI-MS analysis detected the molecular ion of BPA (negatively charged) at *m*/*z* 227, consistent with its molecular mass of 228. We observed no other ion in the mass spectrum, indicating that interfering compounds were not present or were undetectable. The MS/MS analysis confirmed the structure of BPA, with a fragment ion detected at *m*/*z* 212, corresponding to the loss of one methyl group, as well as a fragment ion at *m*/*z* 133, diagnostic of the elimination of the phenol moiety. The NMR analysis showed two doublets at 7.01 and 6.65 ppm and the singlet at 1.57 ppm, characteristic of BPA. The singlet at 1.28 ppm did not belong to BPA; we concluded that it was associated with the presence of an impurity, which we estimated by integration measurements to account for 3 ± 2% of the sample. In addition, in the ^1^H NMR spectrum, the signals between 0.8 and 2.5 ppm corresponding to an impurity (Figure 1B) strongly suggested that this impurity was not a phenolic compound. The degree of purity determined for the BPA used for these experiments (97 ± 2%) was comparable to that reported by the manufacturer (≥ 99%).

### Growth and reproduction parameters

We observed no statistical differences in the dam weights by treatment groups. The percentage of dead pups and the sex ratio were comparable in all groups (Table 1). Additionally, when mated at 2 months of age, the time to first delivery did not differ by treatment group (Table 1).

### Cumulative number of pups per dam

**Figure 2:**
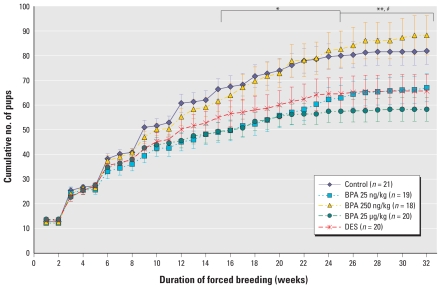
Reproductive capacity of mice exposed perinatally to BPA or 10 ng/kg BW/day DES, expressed as the cumulative number of pups born per dam over a period of 32 weeks of forced breeding (mean ± SE). *n*, number of dams at the beginning of each experiment. Statistical analysis was performed using the Kolmogorov–Smirnov test. For BPA 25 ng/kg and BPA 25 μg/kg compared with controls during the same week, *p < 0.05 for weeks 16–24, and **p < 0.01 for weeks 25–32. For DES compared with controls for the same week, ^#^*p* < 0.05 for weeks 25–32.

The cumulative number of live pups born to BPA 25 ng/kg mice was significantly lower compared with controls starting at week 16 (mean ± SE, 49.74 ± 4.21 pups vs. 67.48 ± 4.38 pups; *p* = 0.037) and persisted until the end of the experiment (67.00 ± 5.70 pups vs. 81.90 ± 5.45 pups; *p* < 0.001) (Figure 2). BPA 25 μg/kg mice also had significantly lower cumulative number of pups per dam compared with control mice. We observed a statistically significant difference between this group and controls beginning at week 16 (49.85 ± 3.69 pups vs. 67.48 ± 4.38 pups; *p* = 0.037) that persisted until the end of the experiment (58.25 ± 4.77 pups vs. 81.90 ± 5.45 pups; *p* < 0.001). The cumulative number of pups born to mice exposed to DES was also significantly lower than in the controls, beginning at week 25 (64.55 ± 6.44 pups vs. 80.00 ± 5.19 pups) and persisting until week 32 (65.70 ± 6.66 pups vs. 81.90 ± 5.45 pups; *p* < 0.01). The cumulative numbers of pups did not differ between the control and BPA 250 ng/kg groups at any time point. However, the BPA 250 ng/kg group differed from the BPA 25 ng/kg and BPA 25 μg/kg groups [77.89 ± 6.64 pups vs. 57.00 ± 4.89 pups (*p* = 0.042) and 56.20 ± 4.19 pups (*p* = 0.042), respectively] starting at week 21 and persisting until the end of the study period [88.22 ± 7.95 pups vs. 67.00 ± 7.70 pups (*p* < 0.001) and 58.25 ± 4.77 pups (*p* < 0.001), respectively]. Likewise, we observed a statistically significant difference between BPA 250 ng/kg and DES groups beginning at week 27 (86.11 ± 7.39 pups vs. 65.45 ± 6.62 pups; *p* = 0.037) and persisting until the end (88.22 ± 7.95 pups vs. 65.70 ± 6.66 pups; *p* < 0.004).

### Fertility

**Figure 3:**
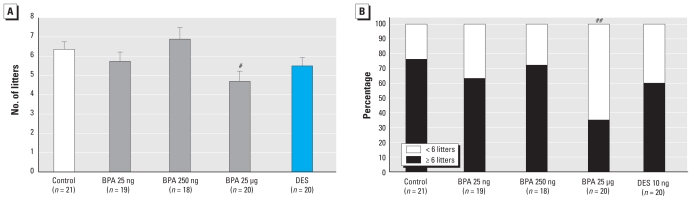
Results after 32 weeks of forced breeding expressed as (*A*) the total number of litters per dam (mean ± SE) and (*B*) percentage of CD-1 female mice per group with greater than or fewer than six litters. *n*, number of dams at the beginning of each experiment. ^#^*p* = 0.024 compared with control, by ANOVA followed by Dunnett’s test. ^##^*p* = 0.012 compared with control, by Fisher’s exact test.

We assessed fertility by the total number of litters per dam for each treatment group at the end of the 32-week period of forced breeding. Dams in the BPA 25 μg/kg group had significantly fewer pregnancies than the control dams (*p* = 0.024) (Figure 3A). We observed a trend toward a decreased number of pregnancies in the BPA 25 ng/kg group (5.74 ± 0.48 pregnancies) and DES group (5.5 ± 0.44 pregnancies) relative to controls (6.37 ± 0.39 pregnancies). The proportion of dams with six or more litters was significantly lower in the BPA 25 μg/kg group (35%) than in the controls (76%) (Figure 3B), as was the proportion of mice with four or more litters in the BPA 25 μg/kg group (60%) compared with the controls (95%; *p* = 0.012).

### Fecundity

**Figure 4:**
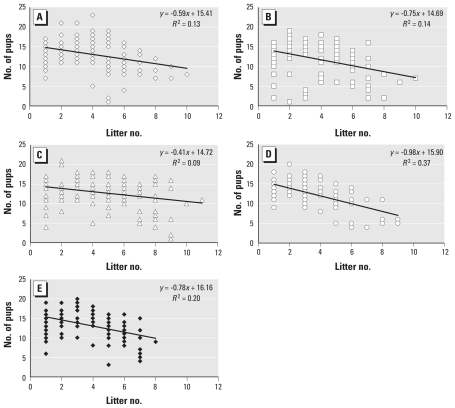
Scatterplot of the number of pups delivered by each dam per litter for control (*A; n* = 21), BPA 25 ng/kg (*B; n* = 19), BPA 250 ng/kg (*C; n* = 18), BPA 25 μg/kg (*D; n* = 20), and DES 10 ng/kg (*E; n* = 22) groups. *n*, number of dams at the beginning of each experiment. Each data point may represent more than one litter because of overlapping data. The slope and the correlation coefficients (*R*) of the trend lines were significantly different for the BPA 25 μg/kg group compared with both the control and BPA 250 ng/kg groups.

We created scatterplots to depict the number of pups born at each delivery for each treatment group, determined a best-fit line for each treatment group, and compared the slopes and correlation coefficients (*R*) of these lines (Figure 4). The difference in correlation coefficients indicated that the variation in fecundity within each treatment group that is attributed to aging differed for the BPA 25 μg/kg group (*R* = 0.60) compared to the control (*R* = 0.36; *p* = 0.025) and BPA 250 ng/kg (*R* = 0.30; *p* = 0.005) groups. The differences in slope indicated that the decline in number of pups delivered over time was hastened in the BPA 25 μg/kg group compared with the control (*p* = 0.012) and BPA 250 ng/kg (*p* = 0.003) groups.

## Discussion

Developmental plasticity has been a central theme in embryology (Gilbert and Epel 2009), and it is currently being explored in medicine and public health because of growing evidence from studies in animal models and humans that various adult-onset diseases, such as obesity, diabetes, and cancer, may have their origins during fetal life (Bateson et al. 2004; Newbold et al. 2008; Soto and Sonnenschein 2010). Exposure to environmental endocrine disruptors during fetal development has been postulated to contribute to declining conception rates and increased incidence of female reproductive disorders such as oocyte aneuploidy, polycystic ovarian syndrome, and altered cyclicity, as well as endometriosis, uterine fibroids, fetal growth retardation, and pregnancy loss (Crain et al. 2008). Perinatal exposure to environmentally relevant doses of BPA has resulted in early vaginal opening and altered estrous cyclicity, overexpression of ERα and progesterone receptor in the endometrium, and an increased number of ovarian antral follicles in mice (Markey et al. 2003, 2005). All of these studies suggest the likelihood of functional consequences such as altered reproductive outcomes in BPA-exposed females.

In the present study, perinatal exposure to BPA resulted in a decline in reproductive capacity in a forced-breeding protocol. BPA 25 μg/kg mice showed a significant decrease in the number of pregnancies, indicating a decline in fertility. We also observed a significant decrease in the cumulative number of pups born to BPA 25 ng/kg, BPA 25 μg/kg, and DES dams relative to controls. The BPA 25 μg/kg group showed a progressive decline in the number of pups delivered, which suggests the possibility of an accelerated reproductive decline. These effects are consistent with previously reported alterations in the ovary, uterus, hypothalamus, and pituitary, the estrogen target tissues that are essential for successful delivery of live pups. Therefore, all these BPA-associated alterations undoubtedly contribute to the diminished reproductive outcome observed here. The forced breeding protocol does not allow for detailed evaluation of fertilization, implantation, and pregnancy maintenance, including the potential for early pregnancy loss in these animals. The protocol records successful pregnancies and provides information about litter size and composition. Therefore, it is impossible to pinpoint a single cause of the reduced fertility observed with perinatal BPA exposure. The overall pattern observed is suggestive of accelerated reproductive decline, which could have involved changes in the function of any or several of the potential BPA target tissues, including alterations at the hypothalamus/pituitary level linked to aging that could have interfered with the preovulatory LH surge and the number of ova ovulated. This study stresses the need to identify the primary targets and the precise sequence of events leading to compromised reproduction.

In contrast to the significant effects on reproductive capacity revealed over time by this forced breeding experiment, neither the time to first delivery nor the number of pups in the first litter was affected by BPA exposure. Standard toxicological studies on BPA and other endocrine disruptors have routinely analyzed the effects on first pregnancy alone, with no follow-up on potential effects in subsequent pregnancies. As a result, they failed to identify changes in reproductive functions that manifest during subsequent pregnancies, leading to an incomplete evaluation of the effects of BPA exposure on fertility. This was pointed out by Honma et al. (2002), who acknowledged that although DES did not affect reproduction in their experiments, it did affect reproductive parameters in prior forced breeding assays. The forced breeding protocol we used in the present study was adapted from McLachlan et al. (1982), who noted a dose-related decrease in reproductive capacity ranging from a decline of the cumulative number of pups in mice exposed prenatally to 10 ng DES/kg BW/day to a high frequency of sterility at the highest dose of 100 μg DES/kg BW/day compared with nonexposed controls. In our study, exposure to 10 ng DES/kg BW/day resulted in a significant decline in the cumulative number of pups born to the treated mice relative to controls.

Of interest, the intermediate BPA dose, 250 ng/kg, did not produce significant effects in the forced breeding regimen, whereas both lower and higher doses did. Natural hormones often show nonmonotonic dose–response curves (Amara and Dannies 1983; Sonnenschein et al. 1989; Vandenberg et al. 2006; Wadia et al. 2007), which have also been reported for several endocrine disruptors (Myers and Hessler 2007). These curves are believed to represent a composite of effects over diverse targets (Vandenberg et al. 2006). Nonmonotonic dose–response curves induced by BPA have been documented in human prostate cancer cell line proliferation (Wetherill et al. 2002), rat pituitary and cerebellar cortex cells (Wozniak et al. 2005), and production of adiponectin by human adipocytes (Hugo et al. 2008). Our data now add reproductive capacity to the list of nonmonotonic responses to BPA exposure. However, it is important to note that although the reproductive capacity of the BPA 250 ng/kg females did not appear to be affected in our forced breeding regimen, it is possible that other experimental protocols would reveal evidence of impaired reproductive function in this treatment group. Indeed, we have observed alterations in estrous cyclicity and antral follicle number (Markey et al. 2003), mammary gland development (Munoz de Toro et al. 2005), and the hypothalamus of females exposed to 250 ng BPA/kg BW/day (Rubin et al. 2006). The forced breeding protocol used here eliminates regular estrous cycles and spontaneous preovulatory LH surges during the 32 weeks of breeding; therefore, it might mask relevant reproductive deficits in this treatment group.

In an earlier study, CD-1 mice exposed to a single bolus injection of a low dose of BPA (25 μg/kg BW) showed significant levels of BPA residues (4% of the dose) in fetuses, confirming the passage of BPA through the placental barrier (Zalko et al. 2003). The finding with regard to the purity of the BPA used in the present study and all our previous studies strongly indicates that it is BPA and not a contaminant that is responsible for the observed effects in exposed offspring. To date, most reports on BPA toxicity rely on the manufacturer’s claim of the purity of the chemical. In the present study we independently assessed and confirmed the purity of the commercial BPA used. In addition, the analysis indicated that the potential for any impurity to correspond to another phenolic compound is negligible. This finding strongly suggests that the effects observed are due to BPA.

Numerous responses to BPA cited above and responses observed in the present study occur with BPA levels that are below the current U.S. Environmental Protection Agency reference dose for this chemical (50 μg/kg BW/day) (Welshons et al. 2003; IRIS-USEPA 2002). In fact, in the present study we used doses that were 2- and 2,000-fold below the current safe dose of BPA. Some of the effects observed at low doses are not found at much higher doses (Myers et al. 2009). Therefore, the current practice in regulatory toxicology of testing chemicals at very high doses might not reliably predict the hazards posed by low doses of endocrine-disrupting chemicals such as BPA.

The current rise in the incidence of infertility cases as well as the advanced onset of puberty in girls may be significantly influenced by the exposure to multiple hormonally active chemicals in our environment. Our findings of reproductive effects in mice after perinatal exposure to low doses of BPA suggest the possibility that environmental exposures to low BPA doses might affect human reproductive health as well and support the need to strictly regulate its use.

## Figures and Tables

**Table 1:** Outcomes of forced reproduction experiment.

Treatment	No. of dams	Total no. of pups		Percent dead pups	Pup sex ratio^a^	Time to first delivery (days)	Dam weight at sacrifice (g)
		Dead	Live				
Control	20	29	1,720	1.66	0.554	21.90 ± 2.61	71.24 ± 3.54
BPA							
25 ng	19	16	1,273	1.24	0.463	22.68 ± 3.87	67.59 ± 2.38
250 ng	18	10	1,588	0.63	0.530	21.22 ± 1.35	72.95 ± 3.05
25 μg	20	26	1,165	2.18	0.506	21.05 ± 1.19	72.93 ± 2.48^b^
DES	20	11	1,501	0.73	0.488	21.73 ± 1.78	78.35 ± 2.51^c^

Values for time to first delivery and dam weight at sacrifice are given as mean ± SE.

a Ratio of males to females.

b Two dams died at 8 months of age, before the end of the study; the cause of the deaths could not be established.

c Three dams died (two dams at 3 months and one dam at 8 months of age) before the end of the study; the cause of the deaths could not be established.
